# The influence of volume and intensive care unit organization on hospital mortality in patients admitted with severe sepsis: a retrospective multicentre cohort study

**DOI:** 10.1186/cc5727

**Published:** 2007-03-22

**Authors:** Linda Peelen, Nicolette F de Keizer, Niels Peek, Gert Jan Scheffer, Peter HJ van der Voort, Evert de Jonge

**Affiliations:** 1 Department of Medical Informatics, Academic Medical Center, Meibergdreef 15, 1105 AZ, Amsterdam, The Netherlands; 2 Department of Anaesthesiology, St Radboud University Medical Center, Department 550, Geert Grooteplein-Zuid 10, 6525 GA, Nijmegen, The Netherlands; 3 Department of Intensive Care, Onze Lieve Vrouwe Gasthuis, Oosterpark 9, 1091 AC, Amsterdam, The Netherlands; 4 Department of Intensive Care, Academic Medical Center, Meibergdreef 9, 1105 AZ, Amsterdam, The Netherlands

## Abstract

**Introduction:**

The aim of the study was to assess the influence of annual volume and factors related to intensive care unit (ICU) organization on in-hospital mortality among patients admitted to the ICU with severe sepsis.

**Methods:**

A retrospective cohort study was conducted using the database of the Dutch National Intensive Care Evaluation (NICE) registry. Analyses were based on consecutive patients admitted between 1 January 2003 and 30 June 2005 who fulfilled criteria for severe sepsis within the first 24 hours of admission. A 13-item questionnaire was sent to all 32 ICUs across The Netherlands that participated in the NICE registry within this period in order to obtain information on ICU organization and staffing. The association between in-hospital mortality and factors related to ICU organization was investigated using logistic regression analysis, combined with generalized estimation equations to account for potential correlations of outcomes within ICUs. Correction for patient-related factors took place by including Simplified Acute Physiology Score II, age, sex and number of dysfunctioning organ systems in the analyses.

**Results:**

Analyses based on 4,605 patients from 28 ICUs (questionnaire response rate 90.6%) revealed that a higher annual volume of severe sepsis patients is associated with a lower in-hospital mortality (*P *= 0.029). The presence of a medium care unit (MCU) as a step-down facility with intermediate care is associated with a higher in-hospital mortality (*P *= 0.013). For other items regarding ICU organization, no independent significant relationships with in-hospital mortality were found.

**Conclusion:**

A larger annual volume of patients with severe sepsis admitted to Dutch ICUs is associated with lower in-hospital mortality in this patient group. The presence of a MCU as a step-down facility is associated with greater in-hospital mortality. No other significant associations between in-hospital mortality and factors related to ICU organization were found.

## Introduction

During the past decade monitoring the performance of health care providers has become common because of increased awareness of accountability and because of increased attention for optimizing quality of care and patient safety [1]. This trend is seen in medicine in general and in intensive care in particular [2,3]. In order to improve the quality of care, patient outcomes in different ICUs are being registered and subsequently compared, with the aim being to identify aspects at the organizational level that influence patient outcome [4,5]. National databases can be a valuable source of information for these comparisons [3].

This report describes a study that made use of a national registry database to investigate the outcomes of patients admitted with severe sepsis to Dutch ICUs. These patients form an important and frequently encountered patient group in the ICU, which is known for its high mortality and consumption of resources [6-9]. It is therefore interesting to compare outcomes in this patient group between ICUs and to seek factors at the ICU level that influence outcome.

This study was conducted to investigate whether variation in risk-adjusted hospital mortality in patients admitted with severe sepsis could be explained by differences in annual sepsis volume or ICU organization.

## Materials and methods

### Patient data

The database of the Dutch National Intensive Care Evaluation (NICE) registry was used in this study. Since 1996, ICUs participating in the NICE registry have provided information on all admissions to those units, with the aim being to assess and compare the performance of the ICUs and to improve the quality of care. Per ICU admission variables are collected that describe patient characteristics, severity of illness during the first 24 hours of ICU admission, and the ICU and in-hospital mortality, and length of stay.

Data collection takes place in a standardized manner according to strict definitions and is subject to stringent data quality checks [10]. This has been shown to ensure that data are of high quality [11]. The data are encrypted such that all patient-identifying information, including name and patient identification number, are removed. In The Netherlands there is no need to obtain consent to make use of registries that do not include patient-identifying information. The NICE initiative is officially registered according to the Dutch Personal Data Protection Act.

The recorded variables are used to calculate probabilities of death for each patient using the Acute Physiology and Chronic Health Evaluation (APACHE) II score [12], the Simplified Acute Physiology Score (SAPS) II [13] and the Mortality Probability Models II [14] at admission and 24 hours. In this study the SAPS II score was used for case-mix adjustment because previous research has shown that this scoring system fits best with the patient population of the NICE registry [15]. Because the organization of ICUs changes over time, data were used from a relatively short and recent period of time, namely all consecutive admissions that took place between 1 January 2003 and 30 June 2005.

### Selection of patients with severe sepsis

Patients were identified as being admitted with severe sepsis if they fulfilled the following criteria within the first 24 hours of ICU admission: confirmed infection with at least two modified Systemic Inflammatory Response Syndrome (SIRS) criteria [16] and at least one dysfunctioning organ system. Precise definitions are given in Table 1. In analogy with the exclusion criteria commonly used in analyses based on the SAPS II scoring system, patients admitted after cardiac surgery, patients admitted with severe burns and patients younger than 18 years were excluded from the analyses. For patients with multiple ICU admissions during a hospitalization period, only the first ICU admission was used [13].

**Table 1:** Definitions used to select patients with severe sepsis at the ICU

Criteria	Definitions used in the study
SIRS criteria	At least two of the following within the first 24 hours of the ICU stay:
	Core temperature > 38.0°C or < 36.0°C
	Heart rate > 90 beats/min
	Respiratory rate = 20 breaths/minute or PaCO_2 _= 32 mmHg or mechanical ventilation
	Leucocyte count < 4,000/mm^3 ^or > 12,000/mm^3^
Infection	Diagnosis of infection confirmed by laboratory results within first 24 hours of ICU stay^a^
Organ	At least one of the following to be present within the first 24 hrs of ICU stay:
dysfunction	Cardiovascular: systolic blood pressure = 90 mmHg or decrease in systolic blood pressure of = 40 mmHg, or use of vasoactive medication to maintain the blood pressure > 90 mmHg for = 1 hour
	Renal: mean urine production < 0.50 ml/kg body weight per hour; if the patient is on chronic renal replacement therapy, then another organ failure dysfunction criterion must be satisfied
	Respiratory: PaO_2_/FiO_2 _= 300 (or PaO_2_/FiO_2 _= 200 if admission diagnosis is respiratory infection)
	Haematological: platelet count = 100,000/mm^3^
	Metabolic: pH = 7.30

^a^In accordance with the definition of 'confirmed infection' used within the NICE registry, a strong suspicion of infection in combination with radiology results (for instance, new infiltrate on thoracic radiograph) and clinical findings (purulent sputum and fever) are also counted as an infection. FiO_2_, fractional inspired oxygen; ICU, intensive care unit; NICE, National Intensive Care Evaluation; PaO_2_, arterial oxygen tension; SIRS, Systemic Inflammatory Response Syndrome.

### Questionnaire

A 13-item questionnaire was developed to obtain information on organizational factors in the ICUs. The questionnaire was developed by a medical informatician and a senior ICU physician. Subsequently, the questionnaire was tested by a panel of six senior ICU physicians involved in the NICE registry who judged the questions to be clear and unambiguous. The questionnaire is provided in Additional file 1.

Information was collected on the size of the ICU and the hospital (expressed as the number of ICU and hospital beds, respectively), the numbers of intensivists and nurses, whether the ICUs had an open or closed format, at which shifts an intensive care physician was exclusively available to the ICU, and the staffing pattern (whether general physicians [doctors temporarily working at the ICU but not in training for specialist status], residents, or fellows in training to become an intensivist formed part of the staff). In previous studies [5,17,18] these variables were found to be related to outcome. Furthermore we asked whether a Medium Care Unit (MCU) was available in the hospital as a step-down facility, with a level of care in between that of the ICU and the general ward; and whether a 24-hour recovery unit was present in the hospital.

The questionnaire was sent to the senior ICU physician responsible for the NICE registry in all ICUs participating in the registry during the study period.

### Statistical analysis

The relationship between volume and organizational factors and in-hospital mortality was assessed using logistic regression analyses.

Before the analyses, the amount of staffing (in full-time equivalents) and the number of ICU beds were used to calculate the 'number of intensivists per ICU bed' and the 'number of nurses per ICU bed'. The annual patient volume and the annual volume of patients admitted with severe sepsis were calculated based on these data. Variables that did not show sufficient variation (defined as > 90% of ICUs providing the same answer) were excluded from the regression analyses.

In the logistic regression analyses, the following modelling strategy was employed. First, to investigate the influence of patient-related factors on in-hospital mortality (which may serve as possible confounders when investigating ICU-related factors), logistic regression analysis was performed using age, sex, SAPS II score and number of dysfunctioning organ systems as covariates. In the remainder of the report this model is referred to as the 'case-mix correction' model. To measure discrimination and calibration of this model, the C index and the Hosmer-Lemeshow C statistic were calculated. Second, logistic regression analyses were performed using in-hospital mortality as the dependent variable and each of the variables related to volume or ICU organization as a covariate, together with the aforementioned possible patient-related confounding factors: age, sex, SAPS II score and number of dysfunctioning organ systems. Third, each covariate with *P *< 0.10 in the preceding analyses was included in a multivariate regression analysis, which was performed using a stepwise backward procedure (with α = 0.05 as a cutoff value).

Continuous covariates (age, SAPS II score, annual number of ICU admissions, annual volume of severe sepsis admissions, number of ICU beds, number of hospital beds, number of intensivists per ICU bed and number of nurses per ICU bed) were included in the models using the fractional polynomials method [19], which makes no assumptions about the functional form of the relationship between the covariate and the outcome. To account for potential correlation of outcomes within ICUs, we used generalized estimation equations with robust variance estimators [20]. A leverage analysis was performed for each of the variables that showed a significant relationship with outcome, to detect whether these results were caused by one single ICU. In this analysis, the leverage of each ICU was determined using a so-called jack-knife approach, which amounts to temporarily removing the data from that of a particular ICU from the dataset and repeating the regression analyses [21]. If the effect of a covariate were to disappear when a particular ICU was excluded from the analyses, then we conjectured that the effect was caused by that particular ICU and therefore should not be considered a general finding. The results from the leverage analysis were also used to verify the confidence intervals for the coefficients of the variables in the full model, because the robust variance estimators in generalized estimation equations are known occasionally to yield confidence intervals that are too wide when the number of clusters is small [22].

The analyses were performed using SPSS version 14.0 (SPSS Inc., Chicago, IL, USA) and SPLUS version 7.0 (Insightful Corp., Seattle, WA, USA).

## Results

During the period of study 32 ICUs were providing data to the NICE registry, thereby covering about one-third of all Dutch ICUs and more than half of all ICU beds in The Netherlands. Twenty-nine of the 32 ICUs returned the questionnaire (response rate 90.6%). One ICU did not register the variable 'confirmed infection', which impeded the selection of severe sepsis patients based on the definition given in Table 1. The remaining 28 ICUs were all located in different hospitals and were all mixed-type ICUs. Three of the units were university affiliated, 20 were teaching ICUs and five were nonteaching ICUs. The four nonresponding ICUs were all mixed-type ICUs, one being university affiliated and three being teaching ICUs.

Together, the responding ICUs admitted 57,765 patients within the study period, of which 23,995 (41.5%) were excluded from the analyses based on the SAPS II criteria, most of them because they were admitted after cardiac surgery. Of the remaining 33,770 patients, 4,605 (13.6%) fulfilled criteria for severe sepsis during the first 24 hours of admission. These patients were included in our study. Table 2 describes characteristics and outcomes of these patients.

**Table 2:** Characteristics and outcome of severe sepsis patients at Dutch ICUs participating in the NICE registry

Characteristic/outcome	Total population of severe sepsis patients (*n *= 4,605)	Interquartile range over ICUs (*n *= 28)
Number of patients with severe sepsis	4,605	90.3–239.5
% of total ICU population	13.6	8.0–16.5
Age (years)	64.1 ± 15.4 (67)^a^	63.1–66.0^b^
Sex (% male)	57.5	55.3–61.6
Severity of illness		
SAPS II score	47.3 ± 17.8 (45)^a^	44.7–48.9^b^
Number of SIRS criteria (%)		
Two	12.8	10.5–16.4
Three	37.5	33.2–39.2
Four	49.6	46.0–54.3
Number of organ dysfunctions (%)		
One	17.2	15.48–23.5
Two	37.0	30.7–41.2
Three	29.1	26.6–32.4
Four	13.1	8.3–15.9
Five	3.6	0.5–5.3
Type of organ dysfunction (%)^c^		
Cardiovascular	88.5	83.8–90.9
Renal	23.7	8.4–32.0
Respiratory	80.4	75.4–82.5
Haematological	23.3	17.4–27.0
Metabolic	33.0	28.4–37.3
Outcome (%)		
ICU mortality	25.0	21.0–30.1
Hospital mortality	34.7	29.3–41.9

Numbers are based on all patients admitted to ICUs participating in the National Intensive Care Evaluation (NICE) registry with severe sepsis between 1 January 2003 and 30 June 2005. Results are presented for the total population (second column), and the interquartile range over the ICUs is given (third column). ^a^Mean ± standard deviation (median). ^b^Mean per ICU. ^c^Percentages do not add up to 100, because a patient can have more than one organ dysfunction. ICU, intensive care unit; SAPS, Simplified Acute Physiology Score; SIRS, systemic inflammatory response syndrome.

The patients admitted with severe sepsis exhibited a higher SAPS II score, as compared with the other ICU patients (mean ± standard deviation: 47.3 ± 17.8 versus 33.3 ± 19.0; *P *< 0.001, by Mann-Whitney *U*-test). Among the severe sepsis patients 1,153 (25.0%) died in the ICU and, in total, 1,599 patients (34.7%) died during hospitalization.

**Figure 1:**
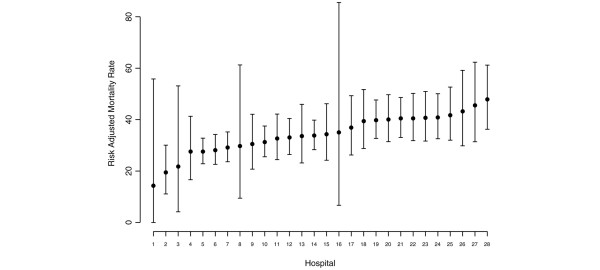
ICU-specific RAMRs for patients admitted with severe sepsis. Values denote the risk-adjusted mortality rate (RAMR) with 95% confidence interval for each of the 28 Dutch intensive care units (ICUs) participating in the study. The RAMR was calculated as follows: based on the case-mix correction model (which included the variables age, sex, SAPS II score, and number of dysfunctioning organ systems), the standardized mortality ratio (SMR) was calculated for each ICU by dividing the observed number of deaths by the number of deaths as expected by the model. The RAMR was subsequently calculated by multiplying the SMR with the overall mean mortality rate in the population of patients admitted with severe sepsis. Values are based on all patients admitted with severe sepsis between 1 January 2003 and 30 June 2005.

The first regression analysis yielded a case-mix correction model in which age, SAPS II score and number of dysfunctioning organ systems were shown to be significantly related to in-hospital mortality. The sex of the patient was not significantly related to outcome, but was retained in the model for case-mix correction purposes. The C index for this model was 0.78 and the Hosmer-Lemeshow C statistic was 1.68 (*P *= 0.99). Figure 1 shows the ICU-specific risk-adjusted mortality rates (RAMRs), along with 95% confidence intervals, based on this case-mix correction model. The RAMR varied between 14.3% and 47.9%.

Table 3 lists the organizational characteristics of the participating ICUs, and the associated odds ratios and *P *values resulting from the regression analyses. Three variables ('resident', 'intensivist responsible for ICU treatment' and 'intensivist exclusively available during weekdays from 07:00 to 18:00 hours') did not show sufficient variability to perform a regression analysis, because 96.4% of the ICUs provided a positive response on these variables. The annual number of patients admitted with severe sepsis and the number of intensivists per ICU bed exhibited significant relationships with hospital mortality (*P *= 0.027 and *P *= 0.036, respectively). The covariate denoting the presence of a MCU as a step-down facility yielded a *P *value of 0.061, and was therefore also included in the multivariate analysis.

**Table 3:** Organizational characteristics of the ICUs and their association with risk-adjusted hospital mortality

ICU characteristic	Descriptive^a^	Odds ratio (95% confidence interval)	*P *value
Number of admissions with severe sepsis per year	72.8 ± 44.0, 65	0.997 (0.995–1.000)	0.027
Total number of admissions per year	522.2 ± 255.0 (488)	1.000 (1.000–1.001)	0.759
Number of ICU beds	15.0 ± 9.8 (12)	0.997 (0.984–1.010)	0.676
Number of hospital beds	561.7 ± 357.6 (510)	1.000 (1.000–1.001)	0.586
Intensivist responsible for ICU treatment	96.4 (27)	^b^	^b^
Intensivist available on weekdays 7–18 hours	96.4 (27)	^b^	^b^
Intensivist available in evening and weekend	67.9 (19)	1.237 (0.878–1.741)	0.224
Number of intensivists per ICU bed	0.30 ± 0.12 (0.33)	1.164 (1.010–1.341)^c^	0.036
Number of nurses per ICU bed^d^	3.6 ± 1.01 (3.8)	0.956 (0.861–1.063)	0.406
Staffing			
General physician^e^	60.7 (17)	0.981 (0.741–1.298)	0.891
Residents	96.4 (27)	^b^	^b^
Fellows in training for intensivist	21.4 (6)	1.014 (0.749–1.374)	0.927
MCU as a step-down facility	42.9 (12)	1.261 (0.990–1.608)	0.061
24-hour recovery in hospital	28.6 (8)	1.118 (0.878–1.425)	0.365

^a^Values are expressed as mean ± standard deviation (median) for continuous variables and percentage (*n*) for dichotomous variables. ^b^Variable not taken into account in regression analysis because of lack of variation. ^c^Odds ratio per 0.1 increase in intensivist-to-bed ratio. ^d^values based on 24 ICUs. ^e^General physician: physician working temporarily at the ICU, not in training for specialist. ICU, intensive care unit; MCU, medium care unit.

The third regression analysis demonstrated significant associations of the annual number of patients admitted with severe sepsis and the availability of a MCU as a step-down unit with in-hospital mortality (Table 4). Admitting a higher number of patients with severe sepsis annually was associated with a lower in-hospital mortality for this patient group. The presence of a MCU as a step-down facility was related to higher in-hospital mortality. Table 5 shows the influence of annual sepsis volume and the presence of a MCU on predicted risk for in-hospital death for a male patient of median age (67 years) and median SAPS II score (45 points) and varying numbers of failing organs. Values are shown for an ICU located at the lower half of the annual sepsis volume (median volume: 38 patients/year) and at the upper half (median volume: 96 patients/year), respectively. In the latter ICU the absolute risk for in-hospital death was 3% to 4% lower than in the ICU admitting 38 patients/year.

**Table 4:** Organizational characteristics that show a significant association with risk-adjusted hospital mortality

Variable	Odds ratio (95% confidence interval)	*P *value
Number of admissions with severe sepsis per year (× 10^-1^)	0.970 (0.943–0.997)	0.029
MCU as step-down facility	1.298 (1.056–1.596)	0.013

Results are based on a multivariate logistic regression analysis. In combination with the risk-adjustment variables, the probability of hospital mortality is calculated as e^(*logit *[*p*])^/(1+ e^(*logit *[*p*])^), where logit(p) = -4.8276 + 0.0601 × SAPS II score + 0.02270 × age -0.02338 × *I*(sex = Female) + 0.01204 × *I*(organ failures = 2) + 0.1257 × *I*(organ failures = 3) + 0.2354 × *I*(organ failures = 4) + 0.3749 × *I*(organ failures = 5) - 0.00306 × annual sepsis volume + 0.2601 *I*(MCU = present). *I *is the identity function, where *I*(x) = 1 if x is true, and *I*(x) = 0 otherwise. SAPS, Simplified Acute Physiology Score; MCU, medium care unit.

**Table 5:** Predicted risk for death for a patient with median characteristics in different organizational settings

Number of failing organ systems	No medium care unit		Medium care unit	
	
	Lower volume quantile^a^	Upper volume quantile^b^	Lower volume quantile^a^	Upper volume quantile^b^
1	29.6	26.0	35.3	31.3
2	29.8	26.2	35.5	31.5
3	32.2	28.4	38.2	34.1
4	34.7	30.8	40.8	36.6
5	37.9	33.8	44.2	39.9

The values show the predicted risk for death for a male patient with severe sepsis of median age (67 years) with a median SAPS II score (45 points) with different values of organ failure, admitted to an ICU at the 50th percentile of the lower and upper volume quantile, respectively, for an ICU with and without a medium care unit as a step-down facility in the hospital. ^a^Annual sepsis volume: 38 patients. ^b^Annual sepsis volume: 96 patients. All values indicate percentages. ICU, intensive care unit; SAPS, Simplified Acute Physiology Score.

The leverage analysis revealed that these findings were not attributable to the influence of individual ICUs. The confidence intervals of the parameters in the full model based on the leverage analysis were comparable to the intervals given in Table 4.

## Discussion

The ICUs participating within the NICE registry showed variation in RAMRs for patients admitted with severe sepsis. We studied factors related to annual volume and ICU organization for this patient group that might explain the variation in RAMRs and found that higher annual volume of patients admitted with severe sepsis was associated with a lower in-hospital mortality in this patient group. The presence of a MCU as a step-down unit increased the probability of in-hospital death for these patients.

The influence of volume on outcome has been studied extensively in other clinical domains. A systematic review [18] found a statistically significant relationship in 70% of the studies that investigated the volume-outcome relationship. A majority of the studies focused on specific (surgical) procedures, such as coronary artery bypass grafting [23] or abdominal aortic surgery [4]. Some previous studies [24,25] investigated the relation between volume and outcome for medical conditions.

Within the area of intensive care, volume-outcome studies have only recently begun to emerge. One study investigated the volume-outcome relation in medical ICU patients [26]. It did not identify a consistent volume-outcome relationship, except for patients admitted with gastrointestinal diagnoses and patients with an Acute Physiology and Chronic Health Evaluation III score above 57 admitted with a respiratory diagnosis.

A recent study [27] found a greater hospital volume to be related to better outcomes in patients who underwent mechanical ventilation. Although this study did not specifically focus on patients admitted to the ICU, and although not all patients admitted to the ICU with severe sepsis undergo mechanical ventilation, evaluation of the findings of that study and those of the present one revealed the presence of similar volume-outcome effects in the two studies. Another study [28] did not find a volume-outcome effect in the general ICU population. However, it was found that hospitals admitting the highest annual numbers of patients at very high risk (SAPS II score > 41) had a significantly lower mortality rate. Our study shows similar findings for patients admitted with severe sepsis (with a mean SAPS II score of 47). All three previous studies were performed in the USA. Our study confirms those findings in Dutch ICU patients admitted with severe sepsis. The previous three studies investigated general groups of patients rather than more specific conditions; by focusing on a specific patient group, we were able to correct further for differences in case-mix.

The results of volume-outcome studies that focused on specific surgical procedures have led to discussions on whether to assign specific procedures to high-volume centres exclusively [29,30]. However, the findings of the present study are not sufficient to support regionalization of ICU care for severe sepsis patients. First, future studies are required to obtain additional evidence of the volume-outcome effect found in the present study. Second, it should be taken into account that, unlike surgical procedures, admission for severe sepsis cannot be planned. Transportation to a high-volume, regionalized severe sepsis centre might do more harm than immediate treatment in a ICU with a low sepsis volume.

Although the volume-outcome effect is a major finding of the present study, the initial focus of the study was not only on the volume-outcome effect, but also on the influence of other organizational ICU characteristics on in-hospital mortality. In recent years several studies have been conducted to investigate the influence of factors related to the organization of the ICU on patient outcomes. Most of these studies were performed in a single ICU. A systematic review that evaluated 26 of these studies [5] found that high-intensity staffing resulted in lower ICU and hospital mortality rates. This was also shown for patients with septic shock in a study that compared mortality rates in these patients during two consecutive periods of staffing, in which the physicians were either trained in critical care medicine or were not [31]. In the present study all but one of the responding ICUs indicated that they employed a closed format, in which the intensivist was primarily responsible for the treatment of the patients. We did not find an association between availability of an intensivist outside working hours and mortality. However, we recognize that this is probably caused by the fact that our data exhibited too little variation to measure an effect, because an intensivist was available round the clock in 74% of the ICUs. With regard to staffing, a study conducted in a medical ICU of a tertiary care medical centre [32] did not find an association between intensivist-to-bed ratio and ICU or hospital mortality rate. In our study we did find an association between intensivist-to-bed ratio and in-hospital mortality when this was the only organizational factor that was taken into account. Unexpectedly, a higher number of intensivists per bed was associated with a higher mortality. In the multivariate analysis the intensivist-to-bed ratio did turn out not to be independently related to in-hospital mortality. For nurse-to-bed ratio no significant relationship with outcome was detected.

The association we found between the availability of a MCU as a step-down unit in the hospital and the risk-adjusted in-hospital mortality is remarkable. Interestingly, no association between availability of a MCU as a step-down unit and hospital mortality was found when ICU mortality was used as an outcome (results not shown). The higher post-ICU mortality in hospitals with a MCU as step-down facility suggests that the changes in organization and staffing that accompany the presence of a MCU do not improve overall patient outcomes. It seems unlikely that transfer to a MCU as a step-down facility *per se *is responsible for a higher mortality.

There are several possible explanations for our findings. First, it could be that ICUs without a MCU transfer their patients to another, better equipped hospital. This would shift the mortality burden from the ICU in a hospital without a MCU to an ICU in a hospital with a MCU. In our study, however, only 150 patients out of 4,605 were transferred to another ICU. When repeating the analyses excluding those patients, similar results were obtained (results not shown). Second, it could be that the presence of a MCU leads to premature patient discharge. Third, we cannot exclude the possibility that, despite our efforts, there are still differences in case-mix that are not taken into account, with the hospitals with a MCU including a patient population with a greater burden of disease. Finally, the availability of a MCU as a step down-unit may act as a confounder for another organizational aspect, possibly unrelated to the MCU, which we did not incorporate in our analyses. To our knowledge the influence of the presence of a MCU as a step-down unit on in-hospital mortality has not previously been specifically investigated. Given our findings, further investigations into the influence of a MCU on patient outcomes are required.

There are some limitations to the present study that must be taken into account when interpreting the results. In the regression analyses we included the patient-specific factors age, sex, SAPS II score and number of dysfunctioning organ systems to account for potential differences in case-mix between ICUs. Although the model solely based on these patient-level factors had good discrimination and calibration, the fit of this model could have been further improved by including other factors (for instance, items relating to chronic disease).

Furthermore, despite the high response rate, the statistical analyses are likely to be influenced by a lack of power. The lack of power might have obscured other possible effects of ICU organization on hospital mortality, which could have been found with a greater number of hospitals. The relatively small number of participating ICUs could have resulted in findings that were dominated by one particular ICU. Several steps were undertaken to reduce this potential problem. First, we did not include variables in the analyses that exhibited too little variation. Second, we used the statistical technique of generalized estimation equations, which compensates for potential correlation of outcomes within ICUs. Finally, the leverage analysis revealed that similar results were obtained based on the jack-knife estimates and that none of the findings were attributable to the influence of individual ICUs participating in the study.

Another limitation of this study is the fact that the questionnaire was sent at the end of the period over which we collected patient data. Because ICU organization changes over time, this might have had a slight influence on the extent to which responses to the survey were representative of the entire study period. To reduce the potential effect of timing of the questionnaire, we used patient data from a 2.5-year period only.

The study was conducted using data from a Dutch national registry, which – at the time of the study – covered about one-third of all Dutch ICUs and more than half of all ICU beds in The Netherlands. The results of our study might not be generalizable to other countries, however, because they may differ in general health care structure, incidence of severe sepsis and availability of treatment strategies. Furthermore, we focused on patients admitted with severe sepsis, and we did not take into account patients who developed severe sepsis while on the general ward or patients who developed severe sepsis after the first 24 hours of ICU stay. Our findings may not apply to those patient groups.

The present study focused only on factors related to ICU organization and did not include treatment aspects. In future analyses, factors related to treatment strategies that are believed to reduce hospital mortality in severe sepsis patients (such as treatments mentioned in the Guidelines from the Surviving Sepsis Campaign [33]) and factors related to limitation of life-sustaining treatment should also be taken into account. Within the NICE registry, however, these data were not available at the patient level.

Finally, in the present study we only focused on part of the treatment period in patients with severe sepsis, namely their stay in the ICU. However, several ICUs responding to the questionnaire indicated that, in their daily experience, outcomes in severe sepsis patients are also influenced by timely recognition of sepsis at the ward, adequate treatment at the emergency department (for instance, use of early goal-directed therapy [34]) and appropriate care after the ICU stay. In the present study these factors were not investigated, but the results, especially the role of the presence of a MCU as a step-down unit, indicate that there is a need for an investigation that takes into account the entire care process for these patients.

## Conclusion

ICUs in the Netherlands exhibit variation in RAMR among patients admitted with severe sepsis. A lower in-hospital mortality in this patient group is associated with a higher number of patients annually admitted with severe sepsis. The presence of a MCU as step-down facility is associated with greater in-hospital mortality. Other associations between in-hospital mortality and factors related to ICU organization were not identified. The volume-outcome effect found in this study must be confirmed by future studies before a change in the admission policy with regard to patients with severe sepsis can be considered.

## Key messages

• ICUs in The Netherlands exhibit variation in RAMR among patients admitted with severe sepsis.

• After adjustment for patient-related factors, a higher annual number of patients admitted with severe sepsis was associated with a lower in-hospital mortality for this patient group.

• The presence of a MCU as a step-down facility was found to be associated with a higher in-hospital mortality.

• The volume-outcome effect found in this study must be confirmed by future studies before a change in admission policy with regard to patients with severe sepsis can be considered.

## Abbreviations

ICU = intensive care unit; MCU = medium care unit; NICE = National Intensive Care Evaluation; RAMR = risk-adjusted mortality rate; SAPS = Simplified Acute Physiology Score.

## Competing interests

During the period from 2002 to 2004 LP received an unrestricted educational grant from Eli Lilly Netherlands BV. The study described in this manuscript was not conducted under this grant, and Eli Lilly Netherlands BV has not been involved in any part of the present study. All other authors declare that they have no competing interests.

## Authors' contributions

LP designed the study, conducted the questionnaire, performed statistical analyses and drafted the manuscript. NdK was involved in the set-up of the study, and helped in interpreting the results and in drafting the manuscript. NP assisted in the statistical analyses, in interpreting the results and in drafting the manuscript. GJS was involved in the set-up of the NICE registry and helped in drafting the manuscript. PvdV was involved in the design of the study and in interpreting the results of the analyses. EdJ participated in the study design, and helped in the design of the questionnaire, in interpreting the results and in drafting the manuscript. All authors read and approved the final manuscript.

## Supplementary Material

Additional file 1 A PDF file including the questionnaire for ICU organization characteristics. Click here for file
