# Supplementary material for: The influence of volume and intensive care unit organization on hospital mortality in patients admitted with severe sepsis: a retrospective multicentre cohort study
Source: Crit Care. 2007 Mar 22;11(2):R40. doi: 10.1186/cc5727 (PMC2206460; doi:10.1186/cc5727)
Supplement: Additional file 1 — A PDF file including the questionnaire for ICU organization characteristics. [file cc5727-S1.pdf]

Appendix 1 Questionnaire for ICU organization characteristics

Question	Response categories	Transformed variable (as used in analyses)
ICU organization		
Is the intensivist primarily responsible for the treatment of the patient?	yes/no	[a]
Is the intensivist exclusively available for the ICU in the following time periods:		
weekdays 7.00h – 18.00h	yes/no	[a]
weekdays 18.00h – 7.00h	yes/no	intensivist available in evening and weekend [b]
weekend	yes/no	
What is the available number of ICU beds?	# of beds	[a]
What is the number of nursing staff?	# of nurses [c]	# of nurses / ICU bed
What is the number of intensivists at your ICU?	# of intensivists [c]	# of intensivists / ICU bed
At your ICU do you have		
general physicians[d]	yes/no	[a]
residents	yes/no	[a]
fellows in training for intensivist	yes/no	[a]
Does your hospital have a MCU as a step-down unit?	yes/no	[a]
Does your hospital have a 24 hour recovery facility?	yes/no	[a]
What is the number of beds in your hospital?	# of beds	[a]

ICU: intensive care unit, MCU: medium care unit; [a] untransformed; [b] the responses for 'intensivist available during evenings' were similar as for 'intensivist available in the weekends'. Therefore these variables have been summarized into one variable. [c]expressed in terms of full time equivalents; [d] Physicians temporarily working at the ICU, not in training for specialist.
